# Accumulation of Microplastics and Potentially Toxic Elements in Plant Leaves Along an Urbanization Gradient in Bangladesh

**DOI:** 10.3390/toxics12120848

**Published:** 2024-11-25

**Authors:** Md. Sohel Parvez, Herta Czédli, Md. Imdadul Hoque, Mohammad Mizanur Rahman, Armin Anwar, Abu Hena Mohammad Mezbah Uddin, Md. Siddiq Hasan, Dina Bibi, Béla Tóthmérész, Tibor Magura, Edina Simon

**Affiliations:** 1 Pál Juhász-Nagy Doctoral School of Biology and Environmental Sciences, University of Debrecen, 4032 Debrecen, Hungary; sohel@nstu.edu.bd; 2 Department of Hydrobiology, University of Debrecen, 4032 Debrecen, Hungary; 3 Department of Oceanography, Noakhali Science and Technology University, Noakhali 3814, Bangladesh; 4 Department of Civil Engineering, University of Debrecen, 4028 Debrecen, Hungary; herta.czedli@eng.unideb.hu; 5 Department of Urdu, University of Dhaka, Dhaka 1000, Bangladesh; s-11th-2016218965@urdu.du.ac.bd; 6 Institute of Marine Sciences, University of Chittagong, Chattogram 4331, Bangladesh; mizan@cu.ac.bd; 7 Department of Environmental Science and Management, North South University, Dhaka 1229, Bangladesh; armin.anwar@northsouth.edu; 8 Department of Botany, University of Dhaka, Dhaka 1000, Bangladesh; mezbah-2014816954@bot.du.ac.bd (A.H.M.M.U.); mdsiddiq-2017514325@bot.du.ac.bd (M.S.H.); 9 Department of Ecology, University of Debrecen, Egyetem tér 1, 4032 Debrecen, Hungary; bibi.dina@science.unideb.hu (D.B.); tothmeresz.bela@science.unideb.hu (B.T.); magura.tibor@science.unideb.hu (T.M.); 10 HUN-REN-UD Functional and Restoration Ecology Research Group, Egyetem tér 1, 4032 Debrecen, Hungary; 11 HUN-REN–UD Anthropocene Ecology Research Group, University of Debrecen, Egyetem tér 1, 4032 Debrecen, Hungary

**Keywords:** microplastics, leaf dust, pollution index, bioindicator, air pollution

## Abstract

Potentially toxic elements (PTEs) and microplastics (MPs) in the atmosphere raise widespread apprehension due to their association with the ecosystem and public health. The accumulation of airborne MPs and PTEs was analyzed in *Polyalthia longifolia* leaves, and the Pollution Index (PI) was calculated along an industrial, residential, and rural gradient in Bangladesh. Only polyethylene terephthalate (PET) was found in the highest concentration in industrial areas compared to other areas. In leaves, a significantly higher Cd, Pb, and Zn concentration was found in industrial regions compared to residential and rural areas. For Cd, the PI was observed to be higher than 1 in rural areas, indicating a moderate level of pollution; it was higher than 3 in residential areas, showing considerable pollution; and it was found to be more than 6 in industrial areas. The higher concentration of both MPs and PTEs with increasing urbanization reflects the influence of anthropogenic activities. The findings of the study demonstrate the fascinating potential of *P. longifolia* tree leaves as a promising bioindicator for air quality biomonitoring.

## 1. Introduction

Air pollution is a serious environmental issue, especially in urban and industrial areas [1,2,3,4]. Air pollutants, through wet and dry precipitation, adversely influence the overall environmental quality by polluting soil and aquatic systems. Air pollution affects not only the ecosystem but also human health [4,5,6]. Various health issues, including lung diseases, might be brought on by even brief exposure to certain air contaminants [7]. Different air pollutants, including airborne plastic particles, particularly nanoplastics (NPs), or, in some instances, microplastics (MPs), may be breathed in and remain in the lungs, causing specific reactions, such as inflammation. In addition, plastic and its additives (dyes and plasticizers) may cause reproductive toxicity, carcinogenicity, and mutagenicity [8]. Plastics are an integral part of today’s society [9]. The daily lives of humans, due to rapid economic development and urbanization, particularly regarding urban textile factories, produce huge amounts of MPs [10]. MPs are widespread in the environment and have sparked significant concern in recent decades due to their multifaceted repercussions on ecosystems [11,12,13]. The consequences of their existence in aquatic and soil ecosystems have been extensively researched, but less focus has been devoted to MP particles in the air [11]; therefore, research is still needed on the incidence, destiny, transit, and impacts of atmospheric MPs [14]. Climate change and weather, such as wind and rainfall, may be impacted by the abundance of MPs in the atmosphere [13]. In general, MPs were found to vary globally from <1 to >1000 pieces m^−3^ in outdoor air, <1 piece m^−3^ in indoor air, from 18 to 225 pieces g^−1^ in outdoor dust, from 10 to 67,000 pieces g^−1^ in indoor dust, and from 2 to 477 pieces g^−1^ in road dust [15]. MPs are constantly discharged into the terrestrial environment and are subsequently carried into the atmosphere by wind and polluted air [16]. MPs can be transported by wind through various means, such as direct emissions and the breakdown of large particles. There have been reports of MPs in the air from various locations and in air masses distant from their inception, indicating their capacity for long-distance transit [12,14]. These airborne MPs can settle on plant and soil surfaces through both wet and dry precipitation [17]. Terrestrial tree canopies could serve as an interim sink for MPs in the atmosphere [18]. Plant leaves have a huge surface area; thus, airborne MPs might potentially be captured there and accumulate to significant quantities over time [19]. In order to evaluate the hazards of inhaling MPs in residential areas, as well as other ecological repercussions on the environment, it is essential to monitor the concentrations of MPs in the atmosphere [20].

Heavy metals are also major contributors to air pollution due to their tenacity in the environment, ability to accumulate in living organisms, and ability to be hazardous and carcinogenic to people despite low doses [1,2]. Because of the higher levels of industry and technology over the last several decades, the rate of emissions of these metals is increasing [21]. These elements, when released into the atmosphere by numerous anthropogenic sources, pose a substantial hazard not only to the environment but also to human health [22]. Respiratory diseases and even mortality can be brought on by exposure to potentially toxic elements (PTEs) [23]. The state and health of the environment are crucial to the overall well-being of mankind [24]. In order to protect both the environment and human health, it is imperative that PTE levels in the air be regularly monitored [1,2]. Earlier studies demonstrated that plants can effectively adsorb and accumulate particles in the atmosphere by trapping atmospheric fine particles on the foliage, for example, foliar dust [25,26,27]. Plant leaves with high coverage, large three-dimensional spaces, and uneven surfaces have the ability to intercept airborne MPs and other particulate matter (PM), acting as an important sink for these pollutants [10,26]. Several studies have indicated that terrestrial plant leaves can trap MPs in the surrounding air [18,28]. It is predicted that 0.13 trillion MPs have adhered to leaf surfaces in the top 11 green countries [18]. Leaves can be used as a bioindicator for environmental status [27,29,30]. Monitoring leaves, irrespective of plant species, can indiscriminately accumulate airborne MPs [18]. Thus, plant leaves can serve as a significant dust-entrapping surface and an effective accumulator of air pollutants, particularly in urban areas and cities [31,32]. Foliage dust could be utilized to trace inorganic pollutants as passive samplers [24,27]. In some cases, for example, in metropolitan areas, using dust deposited on leaves is more advantageous for monitoring the environment than using soil or other parts of plants [31].

We aimed to study air pollution and assess the effect of urbanization based on the accumulation of MPs and PTEs in evergreen tree *Polyalthia longifolia* leaves along an industrial–residential–rural gradient in Bangladesh. We hypothesized that the concentration of both MPs and PTEs is higher along the urbanization gradient in residential and industrial areas than in rural areas. We also hypothesized that the leaves of *P. longifolia* are useful indicators for assessing the level of air pollution.

## 2. Materials and Methods

### 2.1. Study Area and Species

**Figure 1:**
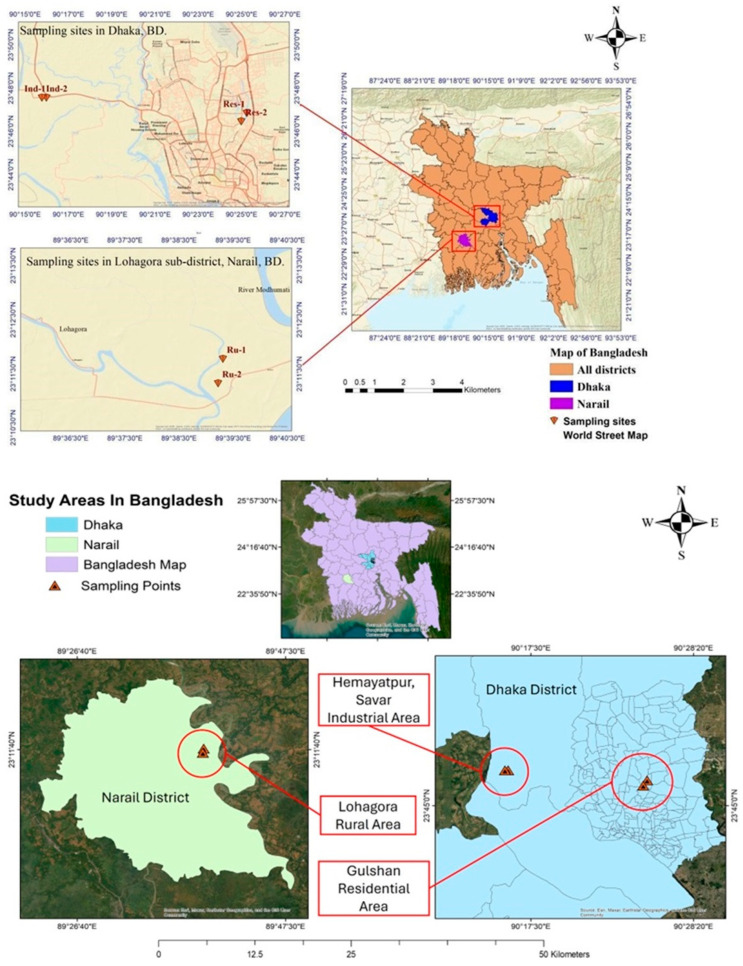
Study areas in Bangladesh (notations: Ind = industrial area, Res = residential area, and Ru = rural area).

Three sampling areas were chosen along an urbanization gradient: an industrial, a residential, and a rural area in Bangladesh (Figure 1). The industrial and residential areas were chosen in the urban region of Dhaka, and the rural area was a rural village. The industrial sampling area was located in an industrial area in Savar (Hemayatpur), Dhaka, with traffic present. Due to its proximity to the capital and the country’s primary commercial and administrative hub, the Dhaka metropolitan area, many industries operate here, the bulk of which are in the textile or garment industry. The residential area was in Dhaka City (Gulshan), with heavy traffic and other anthropogenic activities. Dhaka is a megacity, the most densely populated city in the country, with a population of about 10.2 million, or exactly 10,278,882 persons inhabiting the north and south city corporations [33]. The rural region, on the other hand, was in a village in Bangladesh’s Narail district (Lohagora), where traffic and human activities were low, highlighting the area’s undisturbed, pristine nature. Narail is a district or subdivision under the Khulna division, with a population of only 788,673 [33].

**Figure 2:**
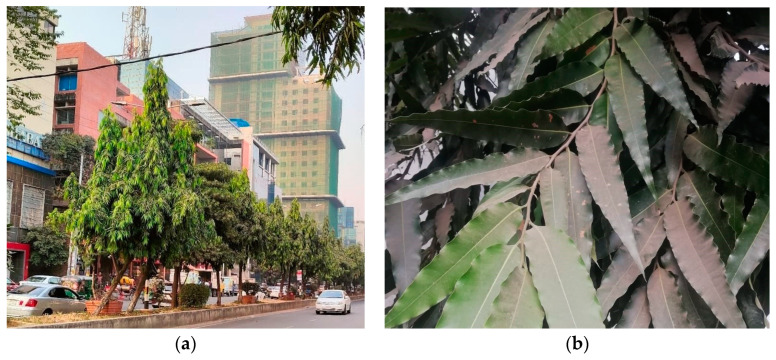
*P. longifolia* trees: (**a**) planted in road dividers in Dhaka city; (**b**) dark/ash coloration indicates dust deposited on leaves.

Leaf samples were collected from *P. longifolia*, locally known as “Debdaru Gach” in Bangla. It is an evergreen tree, widely planted as a roadside and ornamental plant in Bangladesh and other Southeast Asian countries because of its charming, slender, and compact shape and lovely green leaves (Figure 2). Due to their greater opportunity to accumulate pollutants over a more prolonged period, the leaves of evergreen species are regarded as better traps than those of deciduous ones [34]. *P. longifolia* is recognized as a great accumulator of pollutants and is often planted as a barrier against such dangerous compounds [35]. When planted closely together, these trees form a tall natural wall that is used for privacy shielding or ornamental reasons. Therefore, *P. longifolia* was selected as a suitable species for the current research and was also previously studied for pollution appraisals in Bangladesh [35,36] and in other regions [37,38]. Every study area had two locations chosen, and at each site, two individual trees were picked at random. From each tree, 30 leaves were collected in January 2023. Thus, each study region included two pooled samples of a total of 60 leaves, obtained from two individual trees. Samples were collected in paper bags and stored at room temperature in a dark place until analysis.

### 2.2. Quality Assurance and Quality Control (QA/QC)

Quality assurance and quality control (QA/QC) are particularly significant factors for microplastic analyses because of the probability of contamination; hence, sampling and analysis were undertaken with strict attention paid to established quality control protocols. Researchers performing sampling, processing, and analysis for MP research skipped clothes made of synthetic textile materials and instead wore lab coats and apparel made of natural fabrics, such as cotton. Latex gloves made of natural rubber were used, they also replaced gloves frequently. Air circulation was kept as controlled as possible to reduce the spread and contribution of contaminated fibers throughout the laboratory. Before usage, all glassware, stainless-steel filter meshes, scissors, and tweezers were triple rinsed with filtered deionized water. These were thoroughly washed with laboratory detergent and hot water and then rinsed three times with water. Glassware used in processing was dried at a high temperature to remove any leftover plastic residues and stored in locked cabinets or upside down when not in use. A blank was employed throughout the sample preparation and analysis process.

### 2.3. Analysis of Microplastic Concentration

The dust deposited on the collected leaves of *P. longifolia* was washed off the leaves using filtered, deionized water. Filtration was performed using a Whatman glass microfiber filter (47 mm) and a vacuum pump. The leaves were put into a 250 mL glass beaker, and 150 mL of deionized water was added. Then, samples were mixed well by shaking and stirring with a stirrer for 10 min. The dust suspensions were filtered using a 150 μm metal sieve. The leaves were rinsed again with 50 mL of filtered deionized water, which was then filtered and added to the samples. The 200 mL of dust-containing suspensions was transferred onto a hotplate, where its volume was lowered to 25 mL via evaporation at 105 °C. After that, the samples were transferred to previously weighed 100 mL beakers and evaporated totally. The dry weight of the accumulated leaf dust samples was determined by reweighing the beakers containing the samples. Then, 25 mL of filtered, deionized water was added to the samples, transferred to glass tubes, and further analyzed for MPs. Samples were treated with 100 mL 30% (*m*/*m*) hydrogen peroxide overnight. After this, samples were filtered using a sieve (25 mm diameter) with a 0.7 μm glass filter. The concentration of MPs was analyzed by Gas Chromatography–Mass Spectrometry (GC-MS, Trace 1610 with ISQ 7610) with a pyrolyzer (GA/PY-3030D). The program of GC-MS was as follows: 40 °C for 2 min, 280 °C for 10 min, and 320 °C for also 10 min. For the pyrolysis, 600 °C was used, and the time duration was 12 s. A mixture of 11 polymers (Frontier Laboratories) was used for the calibration.

### 2.4. Analysis of Elemental Concentration

After collection, the leaves of *P. longifolia* were washed using tap water since using distilled water might create considerable changes in the concentration of elements in the leaves due to the osmosis process. Drying leaf samples for 24 h at 60 °C was followed by homogenizing the samples using an electrical mixing appliance. The samples were digested for 4 h at 80 °C using 2 mL of 30% (*m*/*m*) hydrogen peroxide and 5 mL of 65% (*m*/*m*) nitric acid; an open digestion system was used [27]. The elemental analysis of the leaf samples was performed using inductively coupled plasma–optical emission spectrometry (ICP-OES 5110, Agilent Technologies, Santa Clara, CA, USA). Peach leaves CRM was used (NIST1547), and the recoveries were under ten percent of the elements’ certified levels (Supplementary Table S1).

### 2.5. Pollution Index

The Pollution Index (PI) was obtained from the proportion of elements detected in the leaves and the background concentration to obtain an overall glimpse of the degree of pollution in each site based on the levels of pollution categories adapted from Faiz et al. [39] and Steindor et al. [40]. The PI of the tree leaves was calculated according to the following equation:

PI = C_sample_/C_n_, (1)

where C_sample_ is the measured levels of the target element in the examined leaves, and C_n_ is the background or reference concentration for that metal in plants recommended by the World Health Organization [41]. The pollution-level categories based on PI values are as follows: PI < 1 = low pollution (LP), 1 ≤ PI < 3 = moderate pollution (MP), 3 ≤ PI < 6 = considerable pollution (CP), and PI ≥ 6 = very high pollution (VHP).

### 2.6. Statistical Analysis

Normality (Shapiro–Wilk test) and homogeneity (Levene’s test) assumptions were confirmed before any statistical analysis of the data. One-way analysis of variance (ANOVA) was performed to examine the differences among variables, followed by Tukey’s HSD (Honestly Significant Difference) post hoc test (*p* < 0.05). The statistical analysis of the data was carried out with SPSS software (Statistical Package for the Social Sciences, 26.0).

## 3. Results and Discussion

### 3.1. Deposition of Microplastics

**Figure 3:**
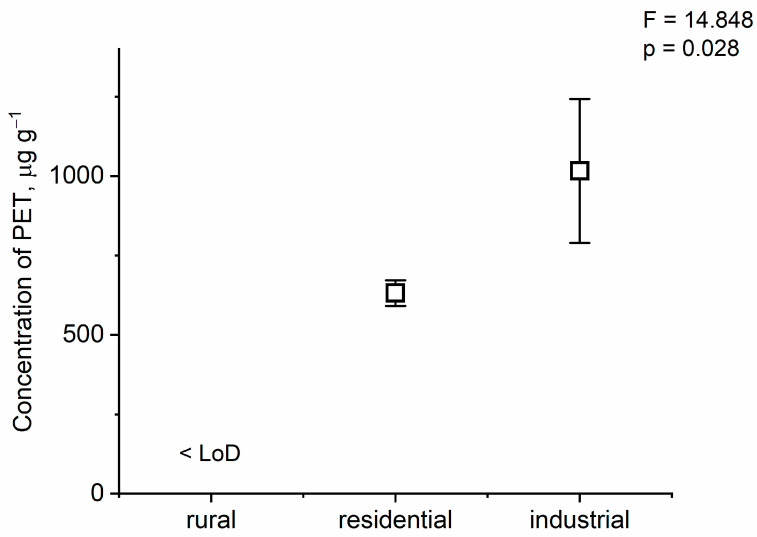
The concentration of PET (mean ± SD). Notation: LoD means the limit of detection.

According to published literature and our knowledge, this study of microplastics in dust deposited on leaves or foliar dust is the first of its type. We detected polyethylene terephthalate (PET)-type MPs in the dust deposited on the leaves of *P. longifolia* in industrial and residential areas. The concentrations of other types of MPs were below the detection limit (µg samples^−1^): polyethylene (PE), <50; polypropylene (PP), <10; polystyrene (PS), <10; acrylonitrile–butadiene–styrene (ABS), <10; styrene–butadiene rubber (SBR), <20; poly methyl methacrylate (PMMA), <10; polycarbonates (PC), <10; polyvinyl chloride (PVC), <20; nylon 6 (N6), <10; and nylon 66 (N66), <10. The concentration of PET varied from 789.47 μg g**^−^**^1^ to 1243.24 μg g^−1^ of dust in the industrial areas and from 591.40 μg g**^−^**^1^ to 672.13 μg g**^−^**^1^ (mean ± SD: 631.76 ± 57.09) of dust in the residential areas in Dhaka, Bangladesh (Figure 3). Significantly higher concentrations were found in the industrial and residential areas than in the rural area (F = 14.848, *p* = 0.028). Higher mean concentrations of MPs were observed in the soils from industries (4.6 ± 4.39 particles kg^−1^) than in the soils near city (1.87 ± 1.81 particles kg^−1^) and urban crop fields (0.58 ± 0.79 particles kg^−1^) in Narayanganj, Bangladesh [42]. Rabin et al. [43] also found the highest concentration of MPs in the street dust of industrial areas (17.33 particles g^−1^), while the lowest was found in that of the residential areas (13.99 particles g^−1^) of Dhaka, as is consistent with the current findings. PET-type MPs and NPs are ubiquitous in the environment, both in atmospheric and airborne forms [44]. PET was reported as the prominent microplastic retained on plant leaves among other airborne MPs [45]. Jafarova et al. [46] detected PET microplastics deposited in *Robinia pseudoacacia* leaflets, alongside other types of MPs. Peng et al. [47] also reported PET and polyamide as two of the most abundant MPs in urban indoor dust. Given their greater density compared to other plastics, PET and polyvinyl chloride MPs have more potential to settle rapidly [16]. PET is a common plastic that is used in many industries, such as textiles, food packaging, beverages, water and juice bottles, automobiles, electronics, construction additives, and more because, of its great chemical and physical properties [45,48,49,50]. Furthermore, its blends are utilized with other thermoplastics and thermosets, making PET an extensively used plastic globally [50]. It is estimated that textile sources account for about 92% of the PET-MPs found in urban dust [47]. PET is one of the most used packaging materials, particularly for the millions of beverage containers and water bottles used every day all around the globe, resulting in the discharge of massive amounts of pollutants into the environment [49,51]. There are huge numbers of industries, mostly textile industries, located around Dhaka. Plastics discharged into the environment from their operations may degrade over time, disperse into the air, and travel through atmospheric circulation. Therefore, the PET-MPs found in the study could have originated from industries operating in the region.

MPs attached to plant leaves were measured by Liu et al. [18] and found to make up to 28% of all deposited compounds. Tree canopies and other aerial particles accumulate air pollutants, based on their particle sizes, wind direction, and velocity [52]. However, the amount of MPs on tree leaves varies depending on leaf type; leaf surface attributes, such as surface roughness; and leaf hydrophilicity [20]. MPs and NPs are typically lipophilic or ‘lipid-loving’ molecules; therefore, they are attracted to the leaf wax layer and, as a result, are absorbed and deposited on the leaf surface [45,53,54]. Tao et al. [55] showed that particles can form C–H···π-type hydrogen bonding with leaf wax. Thus, the PET particles found in the dust deposited on leaves in this study are believed to have been absorbed through this process. There is also a possibility that the nano-PET particles might penetrate the stomata and accumulate in the leaf tissue. It was discovered that 2.5–5 nm graphene quantum dots moved into the mesophyll system of *Platanus acerifolia* leaves, passing through the mechanical barriers in stomata [55]. Wang et al. [56] also observed that MPs can penetrate leaves upon foliar exposure, with stomatal uptake and cuticle entrance being the primary mechanisms for MPs to enter lettuce leaves. Factors including the various tree canopy types, leaf and branch density, and leaf morphology, as well as location and weather circumstances, could affect vegetation’s capacity to retain dust. Airborne particle entanglement is facilitated by leaf trichomes or hairs by expanding the surface area available for accumulating airborne particles and preventing their resuspension [45]. Usually, a tree with rougher and hairier leaves has more potential to capture greater quantities of contaminants than plants with smooth, waxy foliage. Simon et al. [24] observed that trichome density, stomata size, and density were significant factors in dust accumulation and that higher trichome densities could accumulate more dust. The plants growing beside the roadside could accumulate the most dust and contaminants, as evidenced by the work of Liu et al. [31], where they showed the highest level of MPs in pine needles around major traffic routes. However, weather events like rainfall could limit the interception, and intercepting effects diminish as rainfall amounts grow [10]. Through leaf litter or leaf wash-off during precipitation events, MPs deposited on tree canopies can be transported to the soil system [19]. It is also anticipated that the foliage of terrestrial vegetation might operate as a momentary trap and a potential supplier of microplastic contamination in far-off regions [18]. MPs adhered to leaf surfaces may also serve as a layer of absorption for other hazardous materials, fostering the contamination of PTEs or volatile organic compounds on leaf surfaces, and this is equally detrimental to plant health [19]. Sun et al. [25] showed that polystyrene MPs in maize leaves trigger oxidative stress. MPs adhered to leaf surfaces may also obstruct sunlight and impede photosynthesis [19]. PET-MPs can serve as a vector for different airborne pollutants that coexist in the atmosphere, adsorbing via inner or outer adsorption depending on their atomic composition and molecular polarity [44]. Foliage uptake of plastic particles may be a conduit for pollution loading in crop plants, endangering human health [25].

### 3.2. Elemental Concentration of Leaves

Concentrations of Ba, Cd, Co, Cr, Cu, Fe, Mn, Ni, Pb, Sr, and Zn were detected in the leaves of *P. longifolia* (Table 1). The elemental concentrations were found in the order of Fe > Sr > Mn > Zn > Ba > Cu > Pb > Cr > Ni > Co > Cd in the rural area; Fe > Mn > Sr > Zn > Ba > Cu > Pb > Ni > Cr > Co > Cd in the residential area; and Fe > Zn > Sr > Mn > Ba > Cu > Pb > Cd > Cr> Ni > Co in the industrial area. There were significantly (*p* < 0.05) higher concentrations of Cd (0.29 ± 0.08 mg kg^−1^), Pb (5.2 ± 0.14 mg kg^−1^), and Zn (101.4 ± 7.9 mg kg^−1^) in leaves in the industrial area than in those in the residential or rural ones, indicating the influence of industrial operations. This finding is in agreement with the statement that major air-polluting PTEs like Cd and Pb are released mostly from industrial operations [57]. High levels of Cd (0.29 to 2.24 μg g^−1^), Pb (5.37 to 34.90 μg g^−1^), and Zn (11.03 to 30.97 μg g^−1^) were reported in different plant leaves from the industrial site, linked to an iron steel factory, and high levels of Cu (1.06 to 7.39 μg g^−1^) and Al (12.01 to 38.99 μg g^−1^) were found in urban areas [58]. Higher levels of Cd (5 ± 3 ng m^−3^), Pb (118 ± 144 ng m^−3^), and Zn (529 ± 330 ng m^−3^), along with Ni, Mn, and Fe, were observed in the industrial particulate matter than in residential counterparts associated with traffic emission, road dust, the galvanizing and electroplating industry, and the tanning industry [59]. Voutsa et al. [60] also showed that the airborne particulate matter in industrial areas was highly enriched with Cd (0.76–7.8 ng m^−3^), Pb (70–1020 ng m^−3^), and Zn (610–4700 ng m^−3^). Kang et al. [61] reported high airborne concentrations of Pb (206 ± 111 to 554 ± 352 ng m^−3^) and Zn (790 ± 579 to 1112 ± 815 ng m^−3^), along with the presence of Cd (6 ± 4 to 16 ± 14 ng m^−3^), in industrial areas, originating mainly from nonferrous industries. The occurrence of metal(oid)s, Pb (57.0 ± 36.1), and Zn (187 ± 84.7) was observed with Cu (58.5 ± 17.8) in dust coming mainly from vehicle emissions of Dhaka [62]. Shahrukh et al. [36] demonstrated the occurrence of Cd; Pb, along with Cr; and Ni on the tree leaves from anthropogenic origin and concluded that the levels of Pb were unusually high in Dhaka’s atmosphere. More or less similar results were also reported by several researchers, in agreement with our results [29,63,64,65,66,67]. Since they are hubs for human activity, urban and industrial regions are producers of various pollutants [68]. The present study’s industrial sampling site was situated in Savar, Dhaka. It is one of Bangladesh’s most important industrial zones because it is home to the country’s second-largest Dhaka Export Processing Zone and a variety of industries, like textiles, textile washing, printing and dyeing, pharmaceuticals, brickfields, welding, galvanizing, electroplating, etc. [69,70,71]. The emissions and effluents discharged from these industries could be the source of the elevated levels of Cd, Pb, and Zn in the area. The textile industries employ a variety of chemicals for their dyeing and finishing operations, which are linked to the discharge of heavy metals into the environment through wastewater [72]. Different PTEs, including Cd, Pb, and Zn, were reported from textile-industry effluents [72,73], textile dyeing-industry sludge [74,75], pharmaceutical-industry wastewaters [76,77], and electroplating processes [35,78].

However, these PTEs can also be released into the atmosphere from traffic [79,80]. Hazardous elements such as As, Cd, Cr, Cu, Zn, Pb, and Ni are frequently found in trace amounts in particulate matter derived from vehicle emissions [81]. Emissions from traffic were noted as the dominant contributor of Pb, Zn, Mn, and Cr in dust, as well as Pb, Zn, Ni, and Mn in tree leaves, and from industries as the key origin of Cd, Cu, and Ni in dust, and Cd and Cr in tree leaves in Dhaka [35]. Emissions from industrial operations and traffic congestion could be the primary causes of elevated levels of PTEs in the leaves of industrial areas [64]. The Gabtoli bus terminal, which acts as an entry point to and exit point from the capital city from Bangladesh’s northwestern and northern regions, is close to the industrial sampling area, and the Dhaka Aricha highway runs through the area. Therefore, the emissions of a large number of automobiles, as well as industrial operations scattered over the area, make it a highly polluted zone. Salam et al. [82] also reported higher concentrations of Cd in the industrial areas, which they linked to the metal’s release from various industrial mechanical operations. Pb is usually released into the atmosphere through fossil fuel burning, automobile emissions, and home and industrial waste [83]. Leaded fuel used in automobiles contains harmful trace elements, including Pb and Cd [84]. Although, these are banned now and have been replaced with unleaded fuels, the unleaded fuels have been documented to retain a small quantity of Pb, as gasoline may not completely eliminate the presence of Pb [85]. However, the legacy contamination of soil and other parts of the environment due to the widespread use of leaded gasoline before the ban may also operate as a reservoir for residual lead pollution through remobilization and resuspension, which can constantly reintroduce the lead into the atmosphere when disturbed. Moreover, lead smelting and used lead–acid-battery processing and recycling could also be big sources of lead poisoning [86,87]. Cd is frequently utilized in Ni/Cd batteries as a supplementary or rechargeable power source because of its high outcomes, extended lifespan, minimal maintenance needed, and outstanding durability against electrical and physical damage [84]. There is widespread usage of batteries in easy bikes, rickshaws, instant power supply, and solar systems, a majority of which are not adequately recycled or are dumped here and there, thus leaking PB and Cd into the environment [86,87,88]. Increased Zn concentrations are indicative of the role of several contributors to pollution, including heavy vehicular traffic and automobile tire compounds [64]. Zn is emitted mostly by motor vehicles, particularly two-stroke engines, where gasoline and lubricant are mixed and burned together in the piston chambers, with Zn as an ingredient in the lubricating oil, resulting in its emissions during combustion [89]. Zinc may also be released during the manufacturing of galvanized materials, tire wear, and rubber production processes that utilize zinc compounds [90]. The industrial area of the present work is surrounded by numerous brick fields, where bricks are burned using fossil fuels, mostly coal. Different PTEs, including Pb, Cd, etc., exist in trace amounts in coal [91]. Wang et al. [92] demonstrated that the burning of coal, for example, by coal-fired electricity generation facilities, is a major anthropogenic source of PTEs. Coal fly ash was also identified as a source of Dhaka’s atmospheric Cd, Pb, and Zn in earlier studies [93,94]. Thus, coal burning could also be a significant source of Cd, Pb, and Zn in the area. The industrial sampling site (Savar) is geologically characterized by the uplifted Madhupur area covered by dark-reddish-brown-to-brownish-red Madhupur Clay Residuum of the Pleistocene age, backed by the Plio-Pleistocene Dupi Tila sandstone formation [95,96]. The reddish or red soil, formed on karst landforms, usually contains substantial levels of metals, and many studies show that the composition of the parent rock influences the soil’s accumulation of potentially toxic elements [97]. Plant roots can absorb heavy metals from the soil and transfer them to the leaves. Hence, in addition to anthropogenic sources, the nonbiodegradable nature of the hazardous elements and environmental resuspension might also be contributing factors to the metals’ presence in the area. Therefore, the high levels of these PTEs in the industrial site may not originate from a single source but rather could result from several of the different sources discussed above.

We found that the concentrations of Ba, Co, Cr, Cu, Fe, Mn, Ni, and Sr were significantly lower in the industrial zones compared to the rural and residential sites. Simon et al. [98] also found lower levels of Ba, Co, and Cr in urban areas in comparison to rural and suburban locations. The lower levels of these elements in industrial areas may be attributed to the fact that the industries operating in the analyzed region are largely focused on textiles and ready-made garments, which are not as directly associated with these elements as other heavy industries. These industries make the use of effluent treatment plants mandatory, thus substantially reducing the risk of pollution from these sources. Although the other study areas are devoid of extensive industrial activities, there is a huge amount of traffic there. Furthermore, the surroundings of the industrial and residential sites of the present study are currently experiencing several massive development projects, which could be another factor contributing to the elevated levels of contamination in the surrounding area [99]. Therefore, higher levels of these elements in other areas are probably related to growing urbanization and more automobile emissions into the atmosphere. For instance, chromium trioxide (CrO3) in the rubber used in automotive tires and catalytic converters, which can include up to 25 ppm of Cr, is the reason why Shahrukh et al. [36] linked increases in Cr content to the heavy traffic load. There were significantly higher Ba concentrations in the rural and residential areas than in the industrial ones. Molnár et al. [29] also found a higher level of Ba in rural locations than in industrial areas. Although Ba has received little attention in PTE research, it is one of the most harmful PTEs, and all of its derivatives are hazardous [2]. However, the differences between the rural and residential areas for Ba, Cd, Co, Cr, Cu, Fe, Ni, Pb, Sr, and Zn levels were insignificant (*p* > 0.05).

Overall, the high concentration of harmful PTEs with an increasing degree of urbanization found in the study shows the influence of anthropogenic activities, especially industrial operations. Various hazardous materials, including PTEs, are released into the environment as a result of industrial operations and urban activities polluting the nearby areas [100]. Traditionally, such activities revolve around cities or adjacent areas for a number of benefits. Thus, the sharp rise in industrialization and urbanization raises worries globally about the accumulation of PTEs in nature and their impacts on the ecosystem, as well as on public health [101,102]. Additionally, extra fears pertain to the fact that the pursuit of rapid economic growth through industrialization and urbanization might disseminate pollutants, like PTEs, to less inhabited and pristine places [103]. Hence, it is essential to draw attention to the substantial effects of anthropogenic activity on environmental quality and develop mitigation strategies to lower PTE pollution. Regular monitoring of environmental health status using bioindicators could be a significant part of these measures. Plants and soil have more possibility to be the primary sinks for pollutants in urban and industrial regions, especially in cities which are terrestrial habitats. Usually, soil and plant concentrations of different PTEs are highly correlated [104]. There is a positive correlation between PTEs and airborne particulate matter (PM) in soil and plants, denoting the possibility of a transfer of atmospheric and soil PTEs to plants [105]. Therefore, the study of PTEs accumulated in plants via soil from atmospheric deposition could reflect the pollution scenarios in the surroundings. Several researchers also demonstrated the usefulness of plants as a bioindicator of urban environmental status [27,29,34]. Thus, elemental concentration analysis in *Polyalthia longifolia* plant leaves can reflect environmental contamination patterns of PTEs, giving scientific support to tracking air pollution [30].

### 3.3. Pollution Index

**Figure 4:**
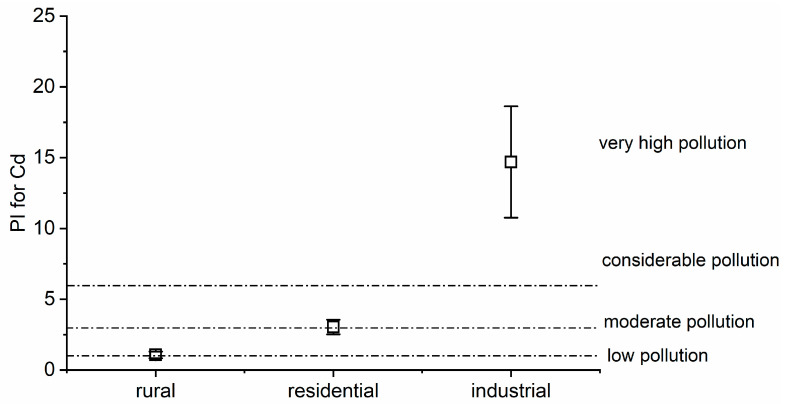
Pollution Index (PI) for Cd in the studied areas (mean ± SD).

**Figure 5:**
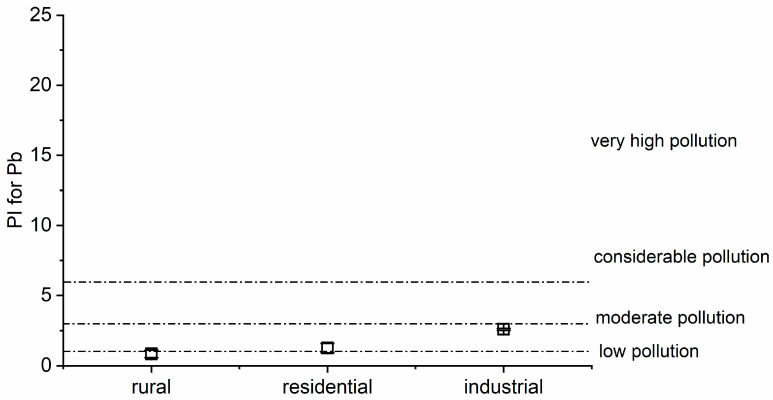
Pollution Index (PI) for Pb in the studies areas (mean ± SD).

The PI indicates the impact of each element at the designated site, which helps assess or continue initiatives aimed at enhancing the condition of the environment by estimating the degree of environmental degradation [35]. The PI of the PTE pollution levels found in the study areas is presented in Supplementary Table S2. The permissible values are Cd = 0.02 mg kg^−1^, for Cr = 1.3 mg kg^−1^, for Cu = 10 mg kg^−1^, for Pb = 2 mg kg^−1^, and for Ni = 10 mg kg^−1^, according to the WHO [41]. For Cd, the PI was observed to be higher than 1 in rural areas, denoting a moderate level of pollution; higher than 3 in residential areas, indicating considerable pollution; and more than 6 in industrial areas, pointing to very high pollution in the vicinity of industrial activities in Dhaka City (Figure 4). Regarding the PI for Pb, the index was lower than 1 in rural areas, signaling a low pollution level, and from 1 to 3 in residential and industrial areas, reflecting moderate pollution (Figure 5). The high PI value for these PTEs could be ascribed to the widespread usage of batteries in easy bikes, rickshaws, instant power supply, and solar systems, a majority of which are not adequately recycled and maintained, leaking PTEs, including PB and Cd, into the environment [86,87,88]. A sizable number of electric vehicles that depend on batteries are operated in Dhaka City each year, but discarded batteries are not properly collected for recycling or are dumped here and there [88]. The high concentration of Pb in Dhaka’s atmosphere might account for the high PI value for Pb seen in residential and industrial regions [36]. In pace with the digital revolution across the globe, the burden of electronic waste (e-waste) is also increasing in Bangladesh. Mowla et al. [106] discovered that inappropriate handling of such material and unofficial e-waste outlets are where PTEs comes from, including Pb, in the metropolis of Dhaka. The presence of tannery enterprises in the vicinity may lead to higher Cd concentrations in the environment [107]. Rahman et al. [108] also reported that Dhaka City is a hot-spot area for Pb, Ni, Cd, and As pollution, which is connected notably with heavy industrial operations. However, there was low contamination (PI < 1) detected in the cases of the other PTEs (Cr, Cu, and Ni). The differences in PI values among the rural, residential, and industrial areas for all the PTEs were found to be significant (*p* < 0.05; Supplementary Table S3). The nature of the element, the type of plant, and the combined impacts of all biotic and abiotic factors generally govern in determining a plant’s capacity to absorb any metal from its surroundings [29]. Plant tissue, species, and PTE types and concentrations all influence plants’ ability to accumulate PTEs [104]. Jashim et al. [109] analyzed the air pollution-tolerance index from different plant leaves in Dhaka City and concluded these to be sensitive to air pollution. Sultan et al. [35] also investigated the PI values for PTEs in different urban areas of Bangladesh, and the results are consistent with the current findings. At the same time, Amadi and Chuku [110] reported that both the translocation factor and bioaccumulation factor were greater than 1 for *P. longifolia*, thus showing that metals were more concentrated in plants, and different parts of plants have the potential to accumulate metals, especially cadmium, lead, and zinc. Kabata-Pendias and Pendias [111] and Turer et al. [112] also demonstrated that plants can accumulate metals from the atmosphere through the leaves, similar to our findings.

## 4. Conclusions

The accumulation of atmospheric MPs on the leaves’ surface and PTEs in the leaf tissue was studied in *Polyalthia longifolia* along an industrial–residential–rural gradient in Bangladesh. The highest levels of MPs and Cd, Pb, and Zn concentrations were obtained in industrial areas, followed by residential and rural areas. The study of the PI for Cd indicated a moderate level of pollution (rural area), considerable pollution (residential area), and very high pollution (industrial area). Meanwhile, in the case of Pb, we found moderate levels of pollution.

Our results also indicated that the polyethylene terephthalate as MPs and the Cd and Pb as PTEs are the main potential environmental risks in the studied areas. Further investigations are needed using other tree species to assess the level of air and soil pollution. The findings demonstrate that *P. longifolia* plant leaves could be a good bioindicator and suitable to be used for biomonitoring in future spatially detailed investigations and/or for tracking changes over time.

## Figures and Tables

**Table 1:** Concentration of PTEs in the leaf samples (mean ± SD, mg kg^−1^). Different letters in the same row indicate significant (*p* < 0.05) differences by Tukey’s HSD test.

Element	Industrial	Residential	Rural	F	*p*
Ba	10.4 ± 0.01 b	20.1 ± 1.20 a	18.2 ± 0.64 a	84.75	0.002
Cd	0.29 ± 0.08 a	0.06 ± 0.01 b	0.02 ± 0.00 b	20.61	0.018
Co	0.06 ± 0.01 b	0.10 ± 0.01 a	0.08 ± 0.01 ab	13.76	0.031
Cr	0.13 ± 0.01 b	0.21 ± 0.02 a	0.18 ± 0.01 ab	22.91	0.015
Cu	5.5 ± 0.15 b	8.5 ± 0.73 a	7.2 ± 0.46 ab	17.56	0.022
Fe	109.5 ± 6.4 b	201.5 ± 28.9 a	147.0 ± 2.8 ab	14.44	0.029
Mn	16.7 ± 3.6 b	48.9 ± 8.8 a	25.6 ± 1.9 b	17.80	0.022
Ni	0.10 ± 0.02 b	0.34 ± 0.08 a	0.15 ± 0.02 ab	13.98	0.030
Pb	5.2 ± 0.14 a	2.5 ± 0.66 b	1.7 ± 0.52 b	27.21	0.012
Sr	29.2 ± 0.6 b	43.9 ± 4.6 a	32.9 ± 2.8 ab	11.83	0.038
Zn	101.4 ± 7.9 a	40.8 ± 10.4 b	25.3 ± 6.9 b	44.32	0.006

## Data Availability

Data could be made available from the corresponding author upon reasonable request.

## Supplementary Materials

The following supporting information can be downloaded at https://www.mdpi.com/article/10.3390/toxics12120848/s1, Table S1: Certified Mass Fraction Values for Elements in SRM 1547; Table S2: Pollution Index values (mean ± SD) in the study areas and the results of ANOVA analysis. Different superscript letters in the same row indicate significant differences (*p* < 0.05) by Tukey’s HSD test.
