# Supplementary material for: Accumulation of Microplastics and Potentially Toxic Elements in Plant Leaves Along an Urbanization Gradient in Bangladesh
Source: Toxics. 2024 Nov 25;12(12):848. doi: 10.3390/toxics12120848 (PMC11679090; doi:10.3390/toxics12120848)
Supplement: Supplementary file 1 [file toxics-12-00848-s001.zip › toxics-3280698-supplementary.pdf]

**Supplementary Materials:**

**Table S1.** Certified Mass Fraction Values for Elements in SRM 1547

| Elements | Certified Values | Recovery% |
|---|---|---|
| Ba | 123.7 | 101 |
| Cd | 0.0261 | 96 |
| Cu | 3.75 | 98 |
| Fe | 219.8 | 93 |
| Mn | 97.8 | 101 |
| Ni | 0.689 | 93 |
| Pb | 0.869 | 91 |
| Sr | 53 | 102 |
| Zn | 17.97 | 98 |

**Table S2.** Pollution index values (mean ± SD) in the study areas and the results of ANOVA analysis. Different superscript letters in the same row indicate significant differences ($p < 0.05$) by Tukey's HSD test.

| Heavy metal | rural | residential | industrial | F | p |
|---|---|---|---|---|---|
| Cd | 1.07 ± 0.23b | 3.04 ± 0.52 b | 14.69 ± 3.93a | 20.62 | 0.018 |
| Cr | 0.14 ±0.01ab | 0.16 ± 0.01a | 0.10 ± 0.01 b | 21.80 | 0.016 |
| Cu | 0.72 ± 0.05 ab | 0.85 ± 0.07a | 0.55 ± 0.01 b | 19.40 | 0.019 |
| Ni | 0.02±0.01 ab | 0.03 ± 0.01a | 0.01 ± 0.01 b | 10.50 | 0.044 |
| Pb | 0.84 ± 0.26 b | 1.24 ± 0.33 b | 2.58 ± 0.07a | 27.92 | 0.012 |
